# Comparing clinical characteristics of systemic sclerosis with or without interstitial lung disease: A cross-sectional study from a single center of the Chinese Rheumatism Data Center

**DOI:** 10.3389/fmed.2022.1061738

**Published:** 2022-12-06

**Authors:** Haoguang Li, Xiuling Zhang, Le Yu, Jingjing Shang, Jie Fan, Xueqin Feng, Rongwei Zhang, Jie Ren, Qifang Guo, Xinwang Duan

**Affiliations:** Department of Rheumatology and Immunology, The Second Affiliated Hospital of Nanchang University, Nanchang, Jiangxi, China

**Keywords:** systemic sclerosis, clinical characteristic, interstitial lung disease (ILD), China, cross-sectional study

## Abstract

**Background:**

We aimed to compare the clinical characteristics of patients with systemic sclerosis (SSc) with or without interstitial lung disease (ILD) to identify relationships with the presence of ILD in SSc at a single center in China.

**Methods:**

A cross-sectional study was conducted using retrospective data from the Chinese Rheumatology Data Center. Patients diagnosed with SSc at the Second Affiliated Hospital of Nanchang University between 2013 and 2022 were included. Demographic and clinical characteristics were compared between patients with SSc with and without ILD. Logistic regression analyses were performed to explore these associations.

**Results:**

A total of 227 patients with SSc were included (male:female ratio = 1:4.82), of which 121 (53.3%) were accompanied with ILD. SSc patients with ILD had a higher percentage of diffuse cutaneous systemic sclerosis (dcSSc), sclerodactyly, loss of finger pad, muscle involvement, left ventricular diastolic dysfunction (LVDD), and pulmonary hypertension (PAH), elevated Krebs von den Lungen-6 (KL-6), and elevated ferritin than those without ILD, and a higher modified Rodnan skin score (mRSS), neutrophil-to-lymphocyte ratio (NLR) and platelet-to-lymphocyte ratio (PLR) (all *P* < 0.05). Antinuclear antibody (ANA) and anti-scleroderma-70 (anti-Scl-70) positivity was presented frequently in SSc patients with ILD, while SSc patients without ILD were more often anti-centromere antibody (ACA) positive (all *P* < 0.05). On the multivariable analysis, muscle involvement [OR 2.551 (95% CI 1.054–6.175), *P* = 0.038], LVDD [OR 2.360 (95% CI 1.277–4.361), *P* = 0.006], PAH [OR 9.134 (95% CI 2.335–35.730), *P* = 0.001], dcSSc [OR 2.859 (95% CI 1.489–5.487), *P* = 0.002], PLR [OR 1.005 (95% CI 1.001–1.008), *P* = 0.020], elevated KL-6 [OR 2.033 (95% CI 1.099–3.763), *P* = 0.024], and anti-Scl-70 [OR 3.101 (95% CI 1.647–5.840), *P* < 0.001] were statistically significant associations with SSc patients with ILD.

**Conclusion:**

Systemic sclerosis was found mainly in females. Several important differences in clinical and laboratory characteristics have been demonstrated between SSc patients with or without ILD. Muscle involvement, LVDD, PAH, dcSSc, PLR, elevated KL-6, and Anti-Scl-70 antibody may be associated with SSc in patients with ILD.

## Introduction

Systemic sclerosis (SSc), also called scleroderma, is a chronic connective tissue disease characterized by localized or diffuse skin thickening and fibrosis. Apart from the skin, SSc also affects internal organs such as the heart, lungs, and gastrointestinal tract, of which interstitial lung disease (ILD) is a common complication, with an incidence ranging from 25–90% (1), and is the major cause of mortality in patients with SSc (2). The interaction between genetic and environmental factors may lead to immune dysfunction, resulting in various immune cells activated and many inflammatory factors produced, in which T helper cells such as Th2 and Th17 are involved in the pathogenesis of SSc (3, 4). Further research revealed that IL-31 and IL-33, novel profibrogenic cytokines produced by activated Th2, may contribute to the development of SSc (5). Besides, Vitamin D, exerting some of its protective effects in the development of autoimmunity through the regulation of Th2 and Th17 cells, was found deficient in SSc and associated with disease activity (6, 7). However, the pathogenesis of SSc has yet to be completely understood. The course of SSc has a wide spectrum, and not all patients have ILD. Furthermore, in clinical practice, treatment is often initiated after clinical symptoms such as dyspnea or cough to develop, pulmonary function decline, or extensive ILD involvement, contributing to intervention delay in SSc-ILD (8). Consequently, identifying patients at high risk of ILD will help to better manage patients with SSc and provide early diagnosis and treatment for SSc-ILD.

Previous studies have confirmed several risk factors for the presence of ILD in SSc, such as male sex, African–American ethnicity, advanced age at time of diagnosis, diffuse cutaneous skin involvement, presence of anti-Scl-70, lower forced vital capacity (FVC), and diffusing capacity of the lung for carbon monoxide (DLCO) (9). Although SSc is widely distributed across the world, the clinical characteristics vary among ethnic and geographic groups (10–12). Previous studies have mainly focused on white populations or Western countries (13–19), so the possible factors related to the presence of SSc with ILD in Chinese patients are not well known. Therefore, we aimed to summarize the clinical characteristics of SSc patients at a single center in China and compare the demographic, clinical, and laboratory characteristics between SSc patients with or without ILD to identify associations with ILD in SSc.

## Materials and methods

### Patients

This cross-sectional study used retrospectively collected data from the Chinese Rheumatology Data Center. Patients diagnosed with SSc at the Rheumatology Department of the Second Affiliated Hospital of Nanchang University from 2013 to 2022 were included in the study. All patients met either the 1980 American Rheumatism Association criteria (20) for SSc or the 2013 American College of Rheumatology/European League Against Rheumatism classification criteria (21) for SSc diagnosis. Patients with symptoms similar to those of SSc (rheumatoid arthritis, polymyositis/dermatomyositis, systemic lupus erythematosus, autoimmune liver disease, vasculitis, or any other CTD except Sjögren’s syndrome) were excluded. All patients were grouped based on whether or not they had ILD according to high-resolution computed tomography (HRCT) manifestations. This study was approved and supervised by the ethics committee of the Second Affiliated Hospital of Nanchang University. Informed consent was obtained from all study participants.

### Data collection

Sex, age at disease onset (since the first non-Raynaud phenomenon symptom), age at first hospital visit, baseline disease duration (from the onset of the first non-Raynaud phenomenon symptom to be classified as SSc), smoking history, body mass index (BMI), and other demographic characteristics were recorded.

Clinical data included SSc-related symptoms and signs (Raynaud phenomenon, sclerodactyly, loss of finger pad, fingertip ulcer, etc.), organ involvement, physician global assessment (PGA), disease type, modified Rodnan skin score (mRSS), chest HRCT, and echocardiography. Patients were categorized as limited cutaneous systemic sclerosis (lcSSc) or diffuse cutaneous systemic sclerosis (dcSSc) using LeRoy’s criteria (22), with systemic sclerosis sine scleroderma considered to be lcSSc. ILD was defined as the presence of alterations on HRCT scans that are consistent with scleroderma-related fibrosis, such as ground-glass opacity, honeycombing, or increased interstitial markings on HRCT scans of the chest. A trained rheumatologist and radiologist independently assessed the images. Another experienced rheumatologist was invited to address the disagreement. The presence of subjective symptoms, including heartburn, reflux, and dysphagia, or reflux esophagitis on gastroscopy or esophageal dilatation on chest CT, was considered as gastroesophageal reflux disease (GERD). Pulmonary hypertension (PAH) was defined as pulmonary artery systolic pressure more than 35 mmHg, according to echocardiography (23). Cardiac involvement is composed of arrhythmia, a decline in left ventricular diastolic dysfunction (LVDD), and pericardial effusion (24). The renal crisis manifests as oliguria, anuria, a progressive increase in serum creatinine, and sudden hypertension (25). Musculoskeletal involvement was also recorded, including muscle damage and arthritis. Arthritis refers to joint tenderness, swelling, or radiographs of the musculoskeletal system showing arthritic changes. Muscle involvement manifested as reduced muscle strength, muscle weakness, and increased creatine kinase levels (CK > 198 U/L), or myogenic damage and myositis according to electromyography and muscle biopsy, respectively (26).

Laboratory tests included white blood cell count (WBC), neutrophil count, lymphocyte count, platelet count, creatine kinase, ferritin (>191 ng/ml was defined as elevated), Krebs von den Lungen-6 (KL-6) (>500 U/mL was defined as elevated), C-reactive protein (CRP) level (>8 mg/L defined as elevated), erythrocyte sedimentation rate (ESR) (>15 and >20 mm/h for males and females was referred to as elevated, respectively), immunoglobulin G (IgG), IgA, IgM, complement 3 (C3), C4, and autoantibody status [antinuclear antibody (ANA), anti-scleroderma-70 (anti-Scl-70) antibody, anticentromere antibody (ACA), anti-u1-ribonucleoprotein (anti-u1-RNP), SSA, SSB, anti-Ro52, rheumatoid factor (RF)]. The neutrophil to lymphocyte ratio (NLR) is the absolute count of neutrophils divided by the absolute count of lymphocytes. Platelet to lymphocyte ratio (PLR) is calculated as the absolute count of platelets divided by the absolute count of lymphocytes.

### Statistical analysis

SPSS for Windows, version 26.0 (IMB Corp., Armonk, NY, USA) was used for data analysis. Kolmogorov–Smirnov test of normality was carried out for measurement data. Normally and non-normally distributed variables were presented as means with standard deviation (SD) and medians with interquartile range (IQR), respectively. Continuous values between groups were compared using the Mann–Whitney test. Chi-squared test or Fisher’s exact test was adopted to compare categorical variables, including proportions, between groups. Univariable and multivariable analyses were carried out logistic regression. Odds ratios (ORs) with 95% CIs were calculated. *P* less than 0.05 were considered statistically significant.

## Results

The baseline demographic and clinical characteristics of 227 patients with SSc are shown in Table 1. The majority of patients (82.8%) were female. The proportion of overweight (BMI > 24) and smokers was 17.2 and 11.9%, respectively. There were 74 cases (34.4%) with dcSSc, 149 cases (65.6%) with lcSSc, and 121 (53.3%) accompanied by ILD. The median age at disease onset and age at diagnosis was 48.2 (IQR 19.4) and 52.6 (IQR 18.3) years respectively, and the median baseline disease duration was 2.1 (IQR 4.4) years. The median mRSS and PGA were 7.0 (IQR, 12.0) and 1.5 (IQR, 1.0), respectively. SSc patients with ILD had a higher percentage of dcSSc than those without ILD (44.6 vs. 22.6%, *P* = 0.001), as did the mRSS [9.0 (IQR 11.5) vs. 5.5 (IQR 13.0), *P* = 0.019].

**TABLE 1:** Comparison of baselines demographics and clinical features between systemic sclerosis with or without interstitial lung disease.

Variables	Total (*n* = 227)	SSc with ILD (*n* = 121)	SSc without ILD (*n* = 106)	*P* *
Female	188 (82.8)	100 (82.6)	88 (83.0)	0.941
BMI (kg/m^2^), median (IQR)	20.9 (3.9)	20.9 (4.4)	20.7 (3.9)	0.448#
BMI > 24	39 (17.2)	24 (19.8)	15 (14.2)	0.257
Age at disease onset (years), median (IQR)	48.2 (19.4)	46.2 (20.0)	49.4 (19.9)	0.562#
Age at first visit (years), median (IQR)	52.6 (18.3)	51.8 (16.7)	53.4 (18.5)	0.412#
Baseline disease duration (years), median (IQR)	2.1 (4.4)	2.3 (4.6)	1.4 (3.6)	0.181#
Smoking history	27 (11.9)	13 (10.7)	14 (13.2)	0.567
mRSS, median (IQR)	7.0 (12.0)	9.0 (11.5)	5.5 (13.0)	0.019#
PGA (0–3), median (IQR)	1.5 (1.0)	1.5 (1.0)	1.2 (1.0)	0.113#
DcSSc	78 (34.4)	54 (44.6)	24 (22.6)	0.001
Raynaud phenomenon	201 (88.5)	106 (87.6)	95 (89.6)	0.634
Fingertip ulcer	65 (28.6)	41 (33.9)	24 (22.6)	0.062
Loss of finger pad	52 (22.9)	34 (28.1)	18 (17.0)	0.047
Telangiectasia	109 (48.0)	51 (42.1)	58 (54.7)	0.059
Sclerodactyly	130 (57.3)	77 (63.6)	53 (50.0)	0.038
Puffy finger	134 (59.0)	69 (57.0)	65 (61.3)	0.511
Finger contracture	17 (7.5)	9 (7.4)	8 (7.5)	0.975
Proximal skin sclerosis	147 (64.8)	83 (68.6)	64 (60.4)	0.196
Arthritis	27 (11.9)	16 (13.2)	11 (10.4)	0.509
Muscle involvement	34 (15.0)	24 (19.8)	10 (9.4)	0.028
GERD	40 (17.6)	25 (20.7)	15 (14.2)	0.199
Cardiovascular involvement	133 (58.6)	81 (66.9)	52 (49.1)	0.006
Arrhythmia	9 (4.0)	6 (5.0)	3 (2.8)	0.632
LVDD	120 (52.9)	74 (61.2)	46 (43.4)	0.007
Pericardial effusion	42 (18.5)	26 (21.5)	16 (15.1)	0.216
Pulmonary hypertension	20 (8.8)	17 (14.0)	3 (2.8)	0.003
Renal crisis	4 (1.8)	2 (1.7)	2 (1.9)	1.000**

Values are the *n* (%) unless otherwise indicated. *Chi-square test was used to compare systemic sclerosis with or without interstitial lung disease groups unless otherwise indicated. **Fisher’s exact test; #Mann–Whitney U-test. SSc, systemic sclerosis; ILD, interstitial lung disease; BMI, body mass index; mRSS, modified Rodnan skin score; PGA, physician global assessment; GERD, gastroesophageal reflux disease; dcSSc, diffuse cutaneous systemic sclerosis; LVDD, left ventricular diastolic dysfunction.

Clinical manifestations included Raynaud phenomenon, found in 201 (88.5%) patients, proximal skin sclerosis in 147 (64.8%), puffy finger in 134 (59.0%), sclerodactyly in 130 (57.3%), telangiectasia in 109 (48.0%), fingertip ulcers in 65 (28.6%), loss of finger pad in 52 (22.9%), and finger contracture in 17 (7.5%). Other organ involvement manifestations comprised of cardiovascular disease (58.6), ILD (53.3%), GERD (17.6%), muscle involvement (15.0%), arthritis (11.9%), PAH (8.8%), and renal crisis (1.8%). Cardiovascular involvement included LVDD found in 120 (52.9%) patients, pericardial effusion in 42 (18.5%), and arrhythmia in 9 (4.0%). In SSc patients with ILD, the prevalence of sclerodactyly, loss of finger pad, muscle involvement, LVDD, and PAH was higher than in those without ILD (63.6 vs. 50.0%, *P* = 0.038; 28.1 vs. 17.0%, *P* = 0.047; 19.8 vs. 9.4%, *P* = 0.028; 61.2 vs. 43.4%, *P* = 0.007; 14.0 vs. 2.8%, *P* = 0.003, respectively). Table 1 presents the baseline demographic and laboratory characteristics of patients at baseline.

Table 2 shows the baseline laboratory features of 227 patients with SSc. The median NLR and PLR were 2.6 (IQR 2.1) and 150.6 (IQR 93.2), respectively. The proportions of SSc patients with increased ferritin, elevated KL-6, elevated CRP levels, and ESR were 21.6, 59.5, 26.4, and 74.9%, respectively. The median CK, IgG, IgA, IgM, C3, and C4 were 83.4 (IQR 86.4), 16.1 (IQR 8.1), 2.7 (IQR 2.0), 1.4 (IQR 0.9), 0.8 (IQR 0.2), and 0.2 (IQR 0.1), respectively. Regarding the autoantibodies, antinuclear antibodies (ANA) were the most common, observed in 78.4% of patients (178/227), followed by anti-Ro-52 (42.3%), anti-Scl-70 (39.6%), RF (32.2%), anti-SSA (31.3%), anti-u1RNP (26.0%), ACA (25.6%), and anti-SSB (6.2%). The median NLR and PLR were higher in SSc patients with ILD than others [2.7 (IQR 2.5) vs. 2.5 (IQR 1.4), *P* = 0.038; 160.0 (IQR 97.3) vs. 139.1 (IQR 79.4), *P* = 0.010, respectively], and SSc patients with ILD had higher proportions of elevated ferritin, elevated KL-6, ANA positivity, and anti-Scl-70 positivity (28.1 vs. 14.2%, *P* = 0.011; 67.8 vs. 50.0%, *P* = 0.007; 86.0 vs. 69.8%, *P* = 0.003; 47.9 vs. 30.2%, *P* = 0.006, respectively), while SSc patients without ILD were more often ACA positive (33.0 vs. 19.0%, *P* = 0.016, respectively).

**TABLE 2:** Comparison of baselines laboratory features between systemic sclerosis with or without interstitial lung disease.

Variables	Total (*n* = 227)	SSc with ILD (*n* = 121)	SSc without ILD (*n* = 106)	*P* *
WBC (×10^9^/L), median (IQR)	5.9 (3.2)	6.4 (3.1)	5.4 (2.5)	0.001#
Neutrophils (×10^9^/L), median (IQR)	3.7 (2.6)	4.1 (2.9)	3.4 (2.0)	0.005#
Lymphocytes (×10^9^/L), median (IQR)	1.4 (0.7)	1.4 (0.7)	1.4 (0.6)	0.920#
Platelet (×10^9^/L), median (IQR)	212.0 (98.0)	238.0 (107.5)	194.5 (104.3)	0.001#
NLR, median (IQR)	2.6 (2.1)	2.7 (2.5)	2.5 (1.4)	0.038#
PLR, median (IQR)	150.6 (93.2)	160.0 (97.3)	139.1 (79.4)	0.010#
CK, median (IQR)	83.4 (86.4)	80.2 (111.6)	84.5 (71.0)	0.881#
Elevated ferritin	49 (21.6)	34 (28.1)	15 (14.2)	0.011
Elevated KL-6	135 (59.5)	82 (67.8)	53 (50.0)	0.007
Elevated ESR	170 (74.9)	92 (76.0)	78 (73.6)	0.671
Elevated CRP	60 (26.4)	37 (30.6)	23 (21.7)	0.130
IgG (g/L), median (IQR)	16.1 (8.1)	16.2 (9.1)	15.8 (6.5)	0.578#
IgA (g/L), median (IQR)	2.7 (2.0)	3.0 (1.9)	2.5 (2.1)	0.438#
IgM (g/L), median (IQR)	1.4 (0.9)	1.4 (0.9)	1.3 (0.9)	0.368#
C3 (g/L), median (IQR)	0.8 (0.2)	0.9 (0.2)	0.8 (0.2)	0.175#
C4 (g/L), median (IQR)	0.2 (0.1)	0.2 (0.1)	0.2 (0.1)	0.245#
ANA	178 (78.4)	104 (86.0)	74 (69.8)	0.003
Anti-Scl-70	90 (39.6)	58 (47.9)	32 (30.2)	0.006
ACA	58 (25.6)	23 (19.0)	35 (33.0)	0.016
Anti-SSA	71 (31.3)	42 (34.7)	29 (27.4)	0.233
Anti-SSB	14 (6.2)	7 (5.8)	7 (6.6)	0.798
Anti-Ro-52	96 (42.3)	58 (47.9)	38 (35.8)	0.066
Anti-u1-RNP	59 (26.0)	32 (26.4)	27 (25.5)	0.867
RF	73 (32.2)	44 (36.4)	29 (27.4)	0.147

Values are the *n* (%) unless otherwise indicated. *Chi-square test was used to compare systemic sclerosis with or without interstitial lung disease groups unless otherwise indicated. #Mann–Whitney U-test. SSc, systemic sclerosis; ILD, interstitial lung disease; WBC, white blood cell; NLR, neutrophil to lymphocyte ratio; PLR, platelet to lymphocyte ratio; CK, creatine kinase; KL-6, Krebs von den Lungen-6; ESR, erythrocyte sedimentation rate; CRP, C-reactive protein; IgG, immunoglobulin G; IgA, immunoglobulin A; IgM, immunoglobulin M; C3, complement 3; C4, complement 4; ANA, anti-nuclear antibody; Anti-Scl-70, anti-scleroderma-70; ACA, anti-centromere antibody; RF, rheumatoid factor; anti-u1-RNP, anti-u1-ribonucleoprotein.

Univariate and multivariate logistic analyses were performed to determine the clinical and laboratory factors associated with ILD in patients with SSc (Table 3). In univariate logistic analysis, loss of finger pad, sclerodactyly, muscle involvement, LVDD, PAH, dcSSc, NLR, PLR, elevated ferritin, elevated KL-6, Anti-Scl-70 antibody, and ACA were statistically significant (*P* < 0.05) (Table 3). The identified variables were subjected to a forward stepwise multiple logistic regression. The multivariable analysis demonstrated that associations with ILD in patients with SSc and muscle involvement [OR 2.551 (95% CI 1.054–6.175), *P* = 0.038], LVDD [OR 2.360 (95% CI 1.277–4.361), *P* = 0.006], PAH [OR 9.134 (95% CI 2.335–35.730), *P* = 0.001], dcSSc [OR 2.859 (95% CI 1.489–5.487), *P* = 0.002], PLR [OR 1.005 (95% CI 1.001–1.008), *P* = 0.020], elevated KL-6 [OR 2.033 (95% CI 1.099–3.763), *P* = 0.024], and Anti-Scl-70 antibody [OR 3.101 (95% CI 1.647–5.840), *P* < 0.001] were statistically significant (Table 3).

**TABLE 3:** Logistic regression analysis of clinical characteristics associated with the presence of ILD in SSc*.

Variables	Univariable analysis		Multivariable analysis	
	Odds ratio (95% CI)	*P*-value	Odds ratio (95% CI)	*P*-value
Loss of finger pad	1.911(1.004−3.636)	**0.049**		
Sclerodactyly	1.750(1.029−2.977)	**0.039**		
Muscle involvement	2.375(1.078−5.233)	**0.032**	2.551(1.054−6.175)	**0.038**
LVDD	2.054(1.208−3.490)	**0.008**	2.360(1.277−4.361)	**0.006**
PAH	5.612(1.596−19.731)	**0.007**	9.134(2.335−35.730)	**0.001**
dcSSc	2.754(1.543−4.914)	**0.001**	2.859(1.489−5.487)	**0.002**
NLR	1.155(1.017−1.312)	**0.027**		
PLR	1.005(1.001−1.008)	**0.008**	1.005(1.001−1.008)	**0.020**
Elevated ferritin	2.371(1.207−4.656)	**0.012**		
Elevated KL-6	2.103(1.227−3.604)	**0.007**	2.033(1.099−3.763)	**0.024**
Anti-Scl-70	2.129(1.232−3.679)	**0.007**	3.101(1.647−5.840)	**< 0.001**
ACA	0.476(0.259−0.875)	**0.017**		

*All variables with a *p*-value below 0.05 from the univariable analysis were entered in multivariable analysis (forward logistic regression). SSc, systemic sclerosis; ILD, interstitial lung disease; LVDD, left ventricular diastolic dysfunction; dcSSc, diffuse cutaneous systemic sclerosis; NLR, neutrophil to lymphocyte ratio; PLR, platelet to lymphocyte ratio; KL-6, Krebs von den Lungen-6; anti-Scl-70, anti-scleroderma-70; ACA, anti-centromere antibody. Bold values indicate *P* < 0.05.

## Discussion

Pulmonary involvement is a destructive complication that affects life expectancy in patients with SSc. In the present study, we summarized the demographic and clinical characteristics of SSc at a single center in China, and compared the differences between SSc patients with and without ILD, with aimed to develop our understanding of ILD in patients with SSc, potentially facilitating early diagnosis, accurate stratification, pre-emptive therapy, and thus improve patient performance (27).

It is well known that SSc is a predominantly female disease, and its peak incidence is between 30 and 50 years of age. Similar to previous studies (28), our data showed that SSc commonly occurs in females, and the median age at disease onset was 48 years. Vascular complications dominate the clinical picture of scleroderma, of which Raynaud phenomenon was the most common initial clinical symptom, with an incidence of 88.5% in this study. In addition, other typical manifestations such as proximal skin sclerosis, puffy finger, sclerodactyly, telangiectasia, fingertip ulcer, and loss of finger pad also prevalently occur in patients with SSc, and this frequency was largely similar in previous studies (29–33). In this study, sclerodactyly and loss of the finger pad occurred more frequently in SSc patients with ILD than in others. Furthermore, a previous study found a correlation between finger pad loss/depressed finger end scars and SSc lung involvement, which aligns with findings from our study (34). Therefore, we speculate that pathological changes in the body surface can reflect internal organ lesions. In addition, the proportion of localized scleroderma in SSc patients was high, accounting for 65.6%, indicating that lcSSc was more common than dcSSc, which is consistent with domestic and international reports (31, 32, 35–42). The differences in clinical patterns and prognosis between patients with dcSSc and lcSSc have been well reported in many previous studies (29, 32, 33). Our study revealed markedly more dcSSc than lcSSc in SSc patients with ILD, which is supported by similar findings from the European Scleroderma Trials and Research (EUSTAR) group and other studies, proving the significance of LeRoy’s criterion (22) in clinical implications.

Renal crisis, the most serious complication of SSc, occurred in 1.8% of SSc patients in this research, consistent with a previous study reporting the renal crisis in the Chinese population to be approximately 1% (33) but lower than that of patients in the Caucasian population (16). In the past 30 years, with the subsequent widespread adoption of angiotensin converting enzyme inhibitors, mortality from the renal crisis has decreased from 42 to 6% (43). Currently, pulmonary complications like PAH and ILD are the major causes of death (2), with incidences of 8.8 and 53.3%, respectively, in this study. A large meta-analysis that included 27 studies with 5,250 participants indicated that the pooled prevalence of ILD among SSc patients in East Asia was 56% (95% CI, 49–63%) (44), which is in line with our study. However, ultrasonic cardiogram examinations were used to estimate pulmonary pressure instead of right heart catheterization, and the thresholds defined by the studies differed, making direct comparisons among studies difficult. In addition, digestive system involvement, mainly GERD, was common in SSc patients, with an incidence of 17.6% in our study, similar to that reported in other Chinese cohorts (29, 31). Moreover, a previous study reported an association between gastroesophageal reflux and ILD in SSc (14, 15), yet these findings were not confirmed in our study.

Cardiac involvement, mainly LVDD, is also frequently seen in systemic sclerosis, found in 52.9% of our SSc population, higher than 31.4%, as found by Hu et al. They adopted more stringent criteria to define LVDD (32). Diastolic dysfunction has been regarded as the outcome of myocardial fibrosis, originating from a coronary microcirculation anomaly and deemed as the pathological hallmark of SSc myocardial disease (45). Previous studies have found that digital pits are significantly more common in SSc patients with LVDD and PAH (46). In addition to the loss of finger pad, LVDD and PAH were significantly associated with ILD, supporting the hypothesis that vascular injury may be one of the common mechanisms for the development and progression of interstitial lung lesions. Interestingly, Zhou et al. revealed that patients with SSc with myopathy tended to present with PAH, ILD, and cardiac involvement (47), which is similar to our results that myopathy was closely related to the presence of ILD in SSc, as revealed by multivariate analysis. Therefore, complex relationships exist among myopathy, PAH, cardiac involvement, and ILD, which require further investigation.

Similar to other connective tissue diseases, SSc is associated with autoantibodies specific to anti-Scl-70 and ACA. Unlike United States, Japan, Australia, and most European countries (35–40), the proportion of anti-Scl-70 (39.6%) antibodies was higher than ACA (25.6%) in this study, which is similar to the frequency of autoantibodies in other Chinese SSc cohorts (12, 31–33). Additionally, the frequencies of anti-Scl-70 and ACA in Chinese patients with SSc are extremely close to that demonstrated by a nationwide multicenter cohort of SSc data from Korea (42.5% Scl-70 and 25.5% ACA) (42). These studies indicate that geography and ethnicity may greatly impact on the occurrence of SSc-associated antibodies. Nevertheless, similar to findings from other ethnic populations (40, 48, 49), the presence of Scl-70 in Chinese patients with SSc was significantly correlated with ILD, whereas ACA was associated with low pulmonary involvement.

Systemic sclerosis-interstitial lung disease is a chronic inflammatory disease involving lymphocytes, neutrophils, and platelets and participates in the regulation of complex inflammatory and immune responses in the organism (50). The NLR and PLR perform well in the assessment of several autoimmune diseases, including rheumatoid arthritis, systemic lupus erythematosus, and so on (51–53). In the present study, PLR, but not NLR, was confirmed to be significantly correlated with the presence of ILD in SSc in multifactorial analysis. In addition, other indicators of inflammation status, such as ferritin, were shown to be higher in SSc patients with ILD than in others in this study, which also supports the above theory. KL-6 is a salivary liquefied glycoprotein, primarily expressed on the surface of type II alveolar epithelial cells, and has been widely confirmed to be correlated with the development and progression of ILD (54–56), similar to the correlation between KL-6 and lung involvement shown in our study.

The advantages of our study are inclusion of a huge number of patients from a single center, thereby reducing possible effects of population heterogeneity. However, some limitations have to be acknowledged in this study. Firstly, it was a cross-sectional study with causality that could not be determined. Second, pulmonary arterial hypertension was estimated indirectly by echocardiography, which may have biased our results. Lastly, this research was carried out in a single rheumatology center, and thus may fail to represent the entire spectrum of SSc in China.

In summary, SSc is characterized mainly by a female predominance. Several important differences in clinical and laboratory characteristics have been observed between patients with SSc with or without ILD. Muscle involvement, LVDD, PAH, dcSSc, PLR, elevated KL-6, and anti-Scl-70 antibody may be associated with ILD in SSc. Future longitudinal cohort studies, including larger populations and longer-term follow-ups, to evaluate cause-effect relationships, are desirable.

## Data availability statement

The datasets used during the current study are available from the corresponding author on reasonable request.

## Ethics statement

The studies involving human participants were reviewed and approved by the Ethics Committee of The Second Affiliated Hospital of Nanchang University. The patients/participants provided their written informed consent to participate in this study.

## Author contributions

XD designed the study and revised the manuscript. XD, HL, XZ, LY, JS, JF, XF, RZ, JR, and QG collected the data. HL performed the data analysis and drafted the first version of the manuscript. All authors read and approved the final manuscript.
